# Inhibitory Activities of *Zygophyllum album*: A Natural Weight-Lowering Plant on Key Enzymes in High-Fat Diet-Fed Rats

**DOI:** 10.1155/2012/620384

**Published:** 2012-11-08

**Authors:** Kais Mnafgui, Khaled Hamden, Hichem Ben Salah, Mouna Kchaou, Mbarek Nasri, Sadok Slama, Fatma Derbali, Noureddine Allouche, Abdelfattah Elfeki

**Affiliations:** ^1^Laboratory of Animal Ecophysiology, Faculty of Sciences of Sfax, University of Sfax, P.O. Box 95, Sfax 3000, Tunisia; ^2^Laboratory of Chemistry of Natural Products, Faculty of Sciences of Sfax, B.P. 1171, Sfax 3000, Tunisia; ^3^Biotechnology High School of Sfax (ISBS), University of Sfax, P.O. Box 261, Sfax 3052, Tunisia; ^4^Hematological and Clinical Biochemistry Laboratory, Hospital of Sidi Bouzid, Sidi Bouzid 9100, Tunisia

## Abstract

Obesity is a serious health problem that increased risk for many complications, including diabetes and cardiovascular disease. The results showed EZA, which found rich in flavonoids and phenolic compounds, exhibited an inhibitory activity on pancreatic lipase *in vitro* with IC_50_ of 91.07 **μ**g/mL. *In vivo* administration of this extract to HFD-rats lowered body weight and serum leptin level; and inhibited lipase activity of obese rats by 37% leading to notable decrease of T-Ch, TGs and LDL-c levels accompanied with an increase in HDL-c concentration in serum and liver of EZA treated HFD-rats. Moreover, the findings revealed that EZA helped to protect liver tissue from the appearance of fatty cysts. Interestingly, supplementation of EZA modulated key enzyme related to hypertension such as ACE by 36% in serum of HFD animals and improve some of serum electrolytes such as Na^+^, K^+^, Cl^−^, Ca^2+^ and Mg^2+^. Moreover, EZA significantly protected the liver-kidney function by reverted back near to normal the values of the liver-kidney dysfunction indices AST&ALT, ALP, CPK and GGT activities, decreased T-Bili, creat, urea and uric acid rates. In conclusion, these results showed a strong antihypelipidemic effect of EZA which can delay the occurrence of dislipidemia and hypertension.

## 1. Introduction

Obesity is a serious health problem worldwide [1, 2]. The prevalence of obesity is rising dramatically among all ages with the changes of lifestyles and dietary fat intake [3]. The accumulation of abnormal or excessive amount of body fat could have several health complications associated with major risk factor for several serious chronic diseases, such as type 2 diabetes, cardiovascular disease, hypertension, stroke, asthma, and certain forms of cancer [4–6]. In fact, higher levels of cholesterol in blood and dietary were considered a major risk factor for coronary heart diseases [7]. Though, lipid lowering could attenuate the progress of these various metabolic disorders and reduce morbidity and mortality [8]. The synthetic pharmacological drugs prescribed against hyperlipidemia and overweight had adverse side effects [9]. Thus, there is a potent need to explore complementary alternative medicine particularly from medicinal plants. One therapeutic approach of obesity and hyperlipidemia is to delay the digestion and absorption of fat via the use of safer lipase inhibitors from natural sources in order to reduce the postprandial hyperlipidemia [10]. *Zygophyllum album *is a shrubby plant belonging to Zygophyllaceae family [11] which includs about 27 genera and 285 species frequently restricted to arid and semiarid areas [12]. In fact, many plants belonging to this genus have anti-inflammatory, molluscicidal, and expectorant activities [13]. The main constituents described from *Zygophyllum* species are zygophyllin, quinovic acid, and glycosides, which have been demonstrated to have anti-inflammatory and antipyretic activity [14]. Furthermore, seven flavonoids together with two phenolic acids were isolated from *Zygophyllum album* L and identified as  quercetin, quercetin-3,7-di-O-*β*-glucopyranoside, isorhamnetin-3-O-*β*-galactopyranoside, isorhamnetin-3-O-*β*-glucopyranoside and isorhamnetin-3-O-*α*-rhamnopyranosyl-(1/6)-O-*β*-glucopyranoside (isorhamnetin-3-O-rutinoside), isorhamnetin-3-O-*α*-rhamnopyranosyl-(1/6)-O-*β*-galactopyranoside (isorhamnetin 3-O-robinoside), isorhamnetin-3-O-*β*-glucopyranoside-7-O-*α*-rhamnopyranoside, gentisic acid, and gentisic acid 5-O-*α*-rhamnopyranoside [14–16]. In fact, *Zygophyllum album* is widespread in the deserts and salt marshes of southern Tunisia. The leaves, stems, and fruits of this plant are used in the Tunisian folk medicine as a drug active against rheumatism, gout, and asthma. It is also used as diuretic, local anaesthetic, antihistaminic, and antidiabetic agent [17]. Therefore, this investigation was aimed to report the therapeutic effect of *Zygophyllum album* against hyperlipidemia and hypertension induced with a standardized high fat-diet (HFD) in female rats along with possible mechanisms.

## 2. Material and Methods

### 2.1. Preparation of Ethanol Extract and Fractions

*Zygophyllum album* was collected from Douz (south of Tunisian) in July 2011. The taxonomic identification of the plant material was confirmed by Professor Mohamed Chaieb in the botany laboratory of the Faculty of Sciences, Sfax University, Tunisia. Air-dried and powdered leaves and flowers (800 g) of *Zygophyllum album* were macerated with 80% ethanol for 24 h three times at room temperature using a mechanical stirrer. The extract was filtered through filter paper and concentrated with a vacuum evaporator. The remaining aqueous solution was fractionated successively with hexane, ethyl acetate, and butanol to obtain the corresponding fractions: hexane (2.7 g), ethyl acetate (69.0 g), and butanol (80.6 g). On the other hand, a second extraction of powdered leaves and flowers (450 g) was performed in the same conditions with 99% ethanol. After filtration, the ethanol was evaporated under reduced pressure to yield the ethanolic extract (45 g).

### 2.2. Determination of Phenolic Content

The total phenolic content in extracts was determined with Folin-Ciocalteau reagent using the method of Chen et al. [18]. A standard curve must be first plotted using gallic acid as a standard. Different concentrations of gallic acid were prepared in methanol, and their absorbances were recorded at 750 nm. 100 *μ*L of diluted sample was added to 2 mL of 2% Na_2_CO_3_ aqueous solution. After 2 min, 100 *μ*L of 50% Folin-Ciocalteau reagent was added. The final mixture was shaken and then incubated at room for 30 min in the dark at room temperature. The absorbance of all samples was measured at 750 nm, and the results are expressed in mg gallic acid equivalents per gram extract (mg GAE/g extract).

### 2.3. Determination of Flavonoid Content

The total flavonoid content in extracts was determined according to Djeridane et al. [19], using a method based on the formation of a complex flavonoid aluminium, having the maximum absorbance at 430 nm. Quercetin was used to make the calibration curve. About 1 mL of diluted sample was mixed with 1 mL of 2% aluminium trichloride (AlCl_3_) methanolic solution. After incubation at room temperature for 15 min, the absorbance of the reaction mixture was measured at 430 nm and the total flavonoid content was expressed in mg quercetin equivalents per gram of extract (mg QE/g extract).

### 2.4. Determination of Lipase Activity *In Vitro*

The method was modified from the assay reported by Nakai et al. [20], in which 4-methylumbelliferyl oleate (4-MU oleate) was used as a substrate to measure the pancreatic lipase inhibitory activity of all samples. Briefly, the assay was conducted by mixing the pancreatic lipase solution with 4-MU solution. The plate was immediately placed in the 37°C preheating FL × 800 micro plate fluorescence reader to measure the amount of 4-methylumbelliferone released by lipase every minute for 30 min at an excitation wavelength of 360 nm with a tolerance of ±40 nm and an emission wavelength of 455 nm with a tolerance of ±20 nm. The lipase inhibitive activity was determined by measuring the effect on the enzyme reaction rate after adding extracts, compared with the control (fluvastin is used as positive control)

(1) PI=Absorbancecontrol−AbsorbancetestAbsorbancecontrol×100.

### 2.5. Animals and Treatments

 The assays of the present study were conducted on adult female *Wistar* rats, weighting 140 ± 10 g, which were obtained from the local Central Pharmacy, Tunisia. All rats were kept in an environmentally controlled breeding room (temperature: 20 ± 2°C; humidity: 60 ± 5%; 12 h dark/light cycle) where they had standard diets and free access to tap water. The experimental protocols were conducted in accordance with the guide for the care and use of laboratory animals issued by the University of Sfax, Tunisia and approved by the Committee of Animal Ethics. The rats were randomly divided in four groups of eight animals each.

Group I: (control) normal female rats were fed with normal chow diet.

Group II: (HFD) female rats received high-fat diet (10% sheep fat + 0.1% cholic acid/kg chow diet) to induce hyperlipidemia for 6 weeks. The cholic acid is a bile acid involved to facilitate the formation of micelles, which promotes processing of dietary fat.

Group III: (fluv.) female rats received HFD and fluvastatin (2 mg/kg, body weight/daily) for 6 weeks. Fluvastatin (trade names Lescol,Canef, and Vastin) is a member of thedrug class of statins, used to treathypercholesterolemia  and to prevent cardiovascular disease.

Group IV: (EZA) female rats received HFD and treated with ethanolic extract of *Zygophyllum album* by gastric gavage route food (400 mg/Kg of body weight/daily) for 6 weeks.

 After the 6 weeks induction, the animals were sacrificed by decapitation in order to minimize the handling stress, and the trunk blood collected. The serum was prepared by centrifugation (1500 ×g, 15min, 4°C), frozen, and stored at −20°C until analysis. The kidney and liver were removed and cleaned of fat. All samples were stored at −80°C until used. For histological studies, pieces of liver were fixed in a Bouin solution for 24 h and then embedded in paraffin. Sections of 5-*μ*m thickness were stained with hematoxylin eosin. The slides were photographed with an Olympus U-TU1X-2 camera connected to an Olympus CX41 microscope (Tokyo, Japan).

### 2.6. Biochemical Analysis

The animals were sacrificed by decapitation and the trunk blood collected. The serum was prepared by centrifugation (1500 ×g, 15 min, 4°C) and stored at −80°C until biochemical analysis. The liver of each rat was excised and homogenized in Tris-Buffered Saline (TBS), pH 7.6 and centrifuged (5000 ×g, 20 min). The supernatant of liver homogenate was frozen and stored for further use in the profile lipid assay. Serum leptin levels were determined by a commercially available Enzyme-Immunoassay (Alpco Diagnostics, Salem NH) which utilizes two specific polyclonal antibodies for mouse and rat leptin. The analyses of serum lipase and serum lipids level of triglycerides (TGs), total cholesterol (T-Ch), high density lipoprotein cholesterol (HDL-c), low density lipoprotein cholesterol (LDL-c) were measured using the corresponding commercial kits (Biolabo, France) on an automatic biochemistry analyzer (BS 300, China) at the pathological laboratory of Sidi Bouzid Hospital. Serum LDL-cholesterol concentration was determined according to formula [21]: LDL_cholesterol_ = total  cholesterol − (triglycerides/5) − HDL_cholesterol_. The serum activity of Angiotensin converting enzyme (ACE) was measured using commercial kit (Trinity Biotech, UK). Serum electrolytes concentrations were determined by an automatic ion analyzer (IA 300, Japan). Serum levels of Creatine phosphokinase (CPK), aspartate aminotransferase (AST), alanine aminotransferase (ALT), alkaline phosphatase (ALP), gamma-glutamyl transpeptidase (GGT) and total bilirubin (T-Bili) activities and creatinine, uric acid, and urea rates were measured in frozen aliquots of serum by standardized enzymatic procedures using commercial kits from (Biolabo, France) on an automatic biochemistry analyzer (Vitalab Flexor E, USA) at the clinic pathological laboratory of Sidi Bouzid Hospital.

### 2.7. Statistical Analysis

Data are presented as means ± standard deviation (SD). Statistical significance was assessed by the Fisher test. *P < 0.05 was considered statistically significant.

## 3. Results

### 3.1. Total Phenolics (TPs) and Total Flavonoids (TFs)

The level of flavonoid and phenolic compounds in different extracts of *Zygophyllum album* is shown in Table 1. Except the hexane extract, which contained low amounts of flavonoid and polyphenol, the other extracts were found to be rich in these compounds. As shown in Table 1, the butanol extract showed the highest amount of phenolic compounds (403.46 mg GAE/g) followed by ethyl acetate (394.28 mg GAE/g) and ethanol (391.65 mg GAE/g) extracts.

### 3.2. Pancreatic Lipase Inhibitory Activity Assay *In Vitro*

As shown in Table 2, for the different solvent extract fractions, the IC_50_ values of ethanol and butanol extracts were (91.07 *μ*g/mL and 94.71 *μ*g/mL), respectively, indicating their strong inhibitory activity over other solvent extract fractions against the pancreatic lipase. It should be noted that fluvastatin showed potent inhibition of pancreatic lipase with a strong IC_50_ = 16.76 *μ*g/mL. The potential inhibitory effects of ethanol extract via other extracts explain the use of this extract for the *in vivo* study.

### 3.3. Effect of EZA on Body Weight of HFD-Fed Female Rats

**Figure 1:**
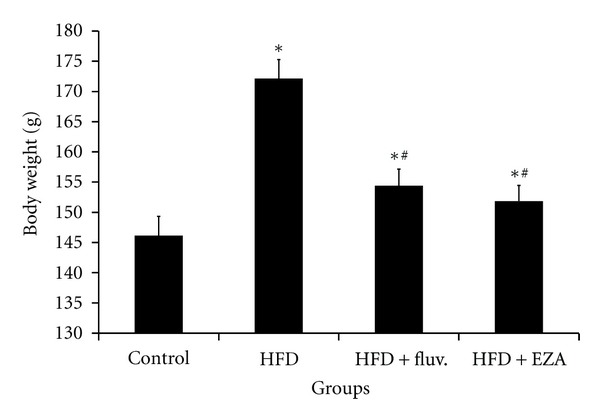
Effect of EZA on body weight of control and HFD treated rats. Values are given as mean ± SD for group of 8 animals each. Values are statistically presented as follows: *P < 0.05 significant differences compared to controls. ^#^ *P* < 0.05 significant differences compared to HFD rats.

By the end of the 6 weeks, the body weight in each group of rats was measured. Also, HFD-fed animals presented significant increase in body by 18% as compared to the control group. However, administration of EZA or fluvastatin to HFD-fed rats reduced significantly the body weight in comparison with the corresponding HFD group by (12 and 10%), respectively, *P* < 0.05 (Figure 1).

### 3.4. Effect of EZA on Serum Leptin Level of Experiment Animals

**Figure 2:**
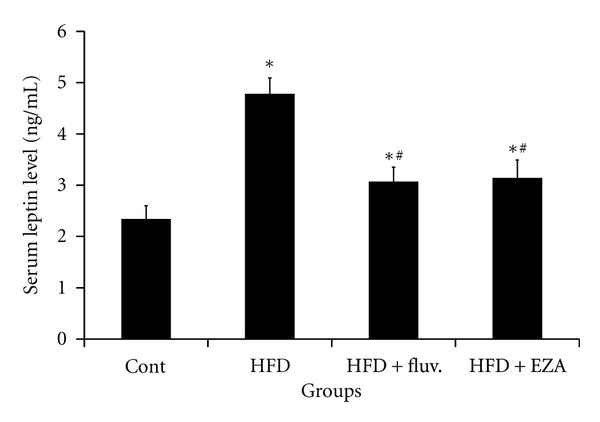
Effect of EZA on leptin level of experimental rats. Values are given as mean ± SD for group of 8 animals each. Values are statistically presented as follows: *P < 0.05 significant differences compared to controls. ^#^ *P* < 0.05 significant differences compared to HFD rats.

Serum leptin level was significantly increased in HFD group by 104% as compared to control animals. However, in response to the fluvastatin and EZA, serum leptin levels in groups received high-fat diet were significantly reduced by 35 and 34%, respectively, as compared to HFD-fed rats, *P* < 0.05 (Figure 2).

### 3.5. Effect of EZA on Lipase Activity and Lipids Profile in Serum and Liver

**Figure 3:**
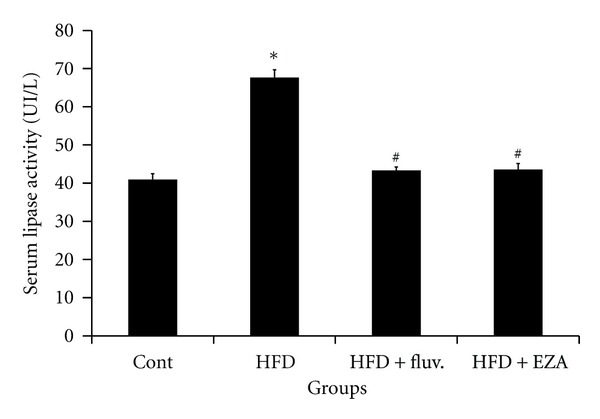
Effect of EZA on lipase activity in serum of control and HFD rats. Values are given as mean ± SD for groups of 8 animals each. Values are statistically presented as follows: *P < 0.05 significant differences compared to controls. ^#^ *P* < 0.05 significant differences compared to HFD rats.

Figure 3 indicated that the lipase activity in serum of HFD-fed animals underwent a potent increase of 65%, as compared to control animals. The rise in lipase activity stimulates lipid absorption and, consequently, led to a remarkable increase in the TC, TG, and LDL-c levels in the serum by 62, 45, and 241%, respectively, and in the liver by 127, 130, and 320%, respectively, associated with significant decrease in the HDL-c level in serum and liver by 18 and 28%, respectively, as compared to the normal female rats. However, the administration of EZA to the obese animals reverted back the lipase activity in serum by 37%, which lowered the rate of TC, TG, and LDL-c and increased HDL-c levels in serum and liver (Table 3). Moreover, fluvastatin supplementation to HFD-fed female rats was observed to improve their lipid profile.

### 3.6. Histopathological Studies of Liver in the Control and Experimental Groups of Rats

**Figure 4:**
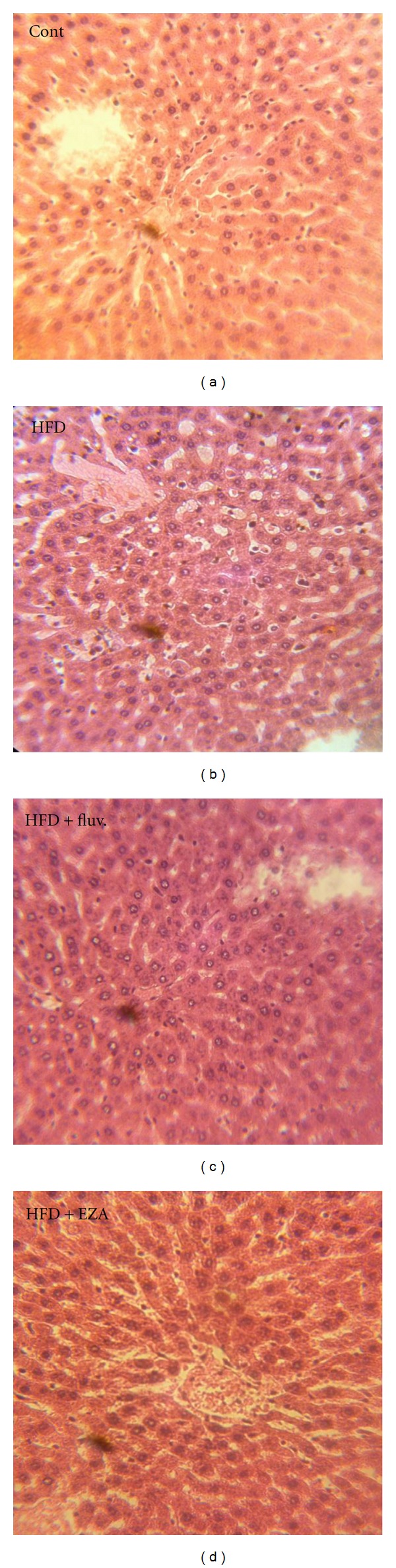
Histopathological studies of liver in the control and experimental groups of rats. The section of the liver from a control rat showing normal architecture; liver of HFD rats showing fatty cysts apparition in liver tissues; the liver of HFD rat treated with fluvastatin or EZA; a potential protective action was shown.

As shown in Figure 4, the section of the liver from a control rat showed a normal architecture. However, liver of HFD rat exhibited fatty cysts apparition in liver tissues, whereas, examination of liver tissue of HFD-fed rats treated with fluvastatin or EZA revealed potential protective action evidenced by reducing the development of fat cells in liver.

### 3.7. Effect of EZA on Serum ACE Activity

**Figure 5:**
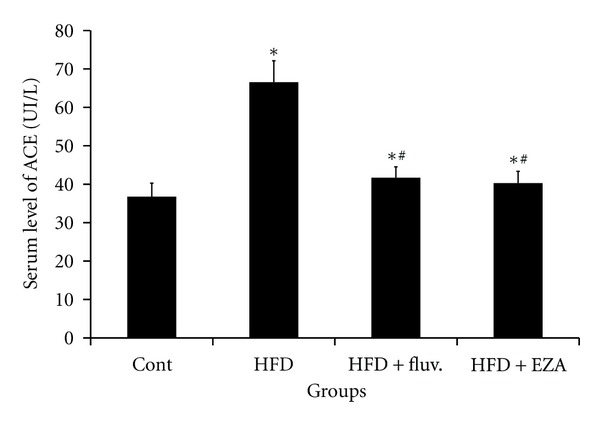
Effect of EZA on serum ACE activity of experimental rats. Values are given as mean ± SD for group of 8 animals each. Values are statistically presented as follows: *P < 0.05 significant differences compared to controls. ^#^ *P* < 0.05 significant differences compared to HFD rats.

Figure 5 evidenced that the ACE activity in the serum of HFD-fed rats underwent a potent increase of 81% as compared to the control rats. However, the administration of the EZA or fluvastatin to the HFD rats was reverted back the activity of ACE in serum back by 36 and 34%, respectively.

### 3.8. Effect of EZA on Serum Major Electrolytes

The findings indicated that compared to the control animals, there were significant reductions in serum Na^+^, Cl^−^, Mg^2+^ and Ca^2+^ by 7, 9, 30, and 22%, respectively, with an increase in the level of K^+^ by 25% (*P* < 0.05), observed in the serum of HFD-fed rats. However, administration of EZA to obese rats reverted back near to normal value of the studied electrolytes (Table 4).

### 3.9. Effect of EZA on Liver-Kidney Function in HFD-Fed Female Rats

Table 5 demonstrated that the HFD-fed rats undertook an increase in terms of the AST, ALT, ALP, CPK, T-Bili, and GGT rates by 82, 50, 123, 42, 51, and 96%, respectively, and creatinine, urea, and uric acid rates in serum by 29, 16, and 20%, respectively, when compared to control rats. Interestingly, the administration of EZA to obese rats seems to have reversed this increase back and ameliorated all indices related to liver and kidney dysfunction induced by high fat-diet.

## 4. Discussion

Obesity is defined as an increase of adipose tissue mass in the body and its accumulation in peripheral organs that leads to metabolic abnormalities such as diabetes mellitus, insulin resistance, and hyperlipidemia [21, 22]. Obesity is thus a worldwide healthcare problem increasing morbid-mortality [22]. Actually, consumption of high-fat diets (HFDs) is a central risk factor for metabolic disorders linked to obesity [23]. Adverse effects of HFD on metabolic homeostasis such as dyslipidemia, diabetes mellitus, and cardiovascular diseases, for example, hypertension and coronary heart disease are linked to adipose tissue physiology and are highly influenced by gender [22–24]. In addition, the use of complementary treatments such as herbal remedies has been considered to have huge potential as an information source and starting point for the development of antiobesity products [25, 26]. *Zygophyllum album* is a deserted plant which belongs to Zygophyllaceae family, widely distributed in southern Tunisia and still used for treatment of diabetes, dermatitis, spasms, and dysmenorrhoea [27]. This study investigated that only EZA potentially inhibited lipase activity with IC_50_ = 91 *μ*g/mL. This inhibitory action of EZA against lipase activity was related to the presence polyphenols and flavonoid compounds in EZA (Table 2). This investigation was in agreement with previous data reporting that alcohol extract of *Zygophyllum album* contains various flavonoid substances such as quercitin-3-O-rutinoside, isorhamnetin-3-O-rutinoside, quinovic acid, kaempherol, Zygophyllin, *β*-sistosterol-*β*-D-glucopyranoside, Carbohydrates, and tannins [13–16]. Previous data reported that the presence of many flavonoids and polyphenol chemical structures was required for the enhancement of pancreatic lipase inhibition [28, 29]. The flavonoid profiles of *Zygophyllum album* and *Zygophyllum decumbens* are very similar whereas that of *Zygophyllum simplex* is quite different. Flavonol 3-O-rutinosides were only present in *Z. album* and *Z. decumbens* and not in *Z. simplex. *However, flavonol 3,7-diglycosides are synthesized by *Z. simplex* and also absent from two other species [30]. *In vivo*, the study revealed that EZA potentially inhibited lipase activity in serum. In fact, the main function of bile acid is to facilitate the formation ofmicelles, which promotes processing of dietary fat digestion by pancreatic lipase [31]. Thus, the pancreatic lipase is secreted from the pancreas, transported to small intestine, and hydrolyzes triglycerides nonabsorbable into absorbable monoglycerides and free fatty acids absorbable by small intestine [32]. It is well known that dietary fat is not directly absorbed from the intestine unless it has been subjected to the action of pancreatic lipase [33]. The application of pancreatic lipase inhibitor was examined as a treatment for diet-induced obesity in humans. It has been clinically reported that a pancreatic lipase inhibitor such as orlistat prevented obesity and hyperlipidemia through the increment of fat excretion into feces and the inhibition of pancreatic lipase [34]. In this study, the inhibition action of EZA on pancreas lipase activity inhibited the hydrolysis of dietary triglycerides nonabsorbable in the intestine into monoglycerides and free fatty acids absorbable by the intestine. This inhibitory action of lipase activity leads to decrease in T-Ch, LDL-c and TGs levels in both plasma and liver, consequently inhibition of lipid accumulation in liver and other tissues such as muscle consequently decrease in body weight as antiobesity action [35]. This increase of lipid accumulation in various tissues of high-fat diet rats is associated with elevated leptin level [36]. This result in accordance with the study of Wang et al. [37], reported that leptin level is directly correlated to adipocyte lipid accumulation and hence, circulating leptin level is an ideal indicator of assessing obesity in both experimental animals and humans [38]. The positive effect of EZA on obesity and hyperlipidemia in both serum and liver was confirmed by a significant decrease of serum leptin level as compared to HFD rats. Moreover, the decrease of leptin level in serum of rats treated with EZA can prevent many other disorders related to obesity and hyperlipidemia such as hypertension. In fact, several previous studies revealed a strong correlation between serum leptin levels and body fat mass, suggesting that there is a leptin-resistant mechanism in obesity [39, 40]. In addition, to regulate body fat mass, leptin also have multiple complex actions on cardiovascular and renal systems, such as sympathetic activation, increased insulin sensitivity, and renal sodium and water excretion [41]. Chronic effects of leptin increased arterial pressure despite a decrease in food intake. The rise of heart rate and renal vascular resistance were also noted and explained the consistent sympathetic activation by leptin [42]. Our findings showed that HFD-fed rats exhibited a potent increase in serum leptin associated with a significant rise in angiotensin converting enzyme activity (ACE), key enzyme related to hypertension. Hence, obesity is wide associated with the overexpression of ACE [43–45]. Interestingly, our study revealed that EZA modulated ACE activity in serum of HFD-fed rats. Actually, in the treatment of hypertension, inhibition of the angiotensin converting enzyme is established as a one modem therapeutic principle. Other major drugs to treat hypertension are *β*-receptor blockers and calcium antagonists. Further, supplementation of EZA improves some of serum electrolytes involved in blood pressure and obesity homeostasis such as Na^+^, K^+^, Cl^−^, Ca^2+^, and Mg^2+^. This result in accordance with previous observations that high dietary Calcium and Magnesium both with and without dairy reduce WAT inflammatory gene expression was confounded by Ca/dairy-associated reductions in body weight and adiposity [46, 47]. They proved remarkably efficient in the decrease of liver and kidney dysfunction indices in HFD-fed rats, namely, the AST, ALT, ALP, CPK, T-Bili, and GGT rates and the urea and creatinine levels. The strong therapeutic effect and potential of EZA are presumably associated with its potent antiobesity and hypolipidemic properties.

## 5. Conclusion

The results demonstrated a strong antihypelipidemic effect of EZA which can delay the occurrence of dislipidemia and hypertension complications. Thus, this study provides the possible pharmacologic rationale to the medicinal use of *Zygophyllum album* in the development of antiobesity drugs.

## Figures and Tables

**Table 1:** Flavonoid and total phenolic contents of various extracts of  *Zygophyllum album*.

Sample	Total flavonoid content (mg QE/g extract)	Total phenolic content (mg GAE/g extract)
Hexane fraction	21.8 ± 0.11	78.022 ± 1.56
Ethyl acetate fraction	47.16 ± 0.23	394.28 ± 7.88
Butanol fraction	45.8 ± 0.23	403.46 ± 8.06
Ethanolic extract	40.98 ± 0.20	391.65 ± 7.83

**Table 2:** *In vitro* pancreatic lipase inhibition assay of various extract of  *Zygophyllum album*.

Sample	Concentration (*μ*g/mL)	% inhibition	IC_50_ (*μ*g/mL)
	25	74.58 ± 1.13	
Fluvastatin	50	86.78 ± 1.27	16.76
	100	93.35 ± 1.25	

Hexane extract	50	33.43 ± 1.11	
	100	48.52 ± 1.42	103.05
	200	59.85 ± 1.08	

Ethyl acetate	50	36.88 ± 1.45	
	100	46.23 ± 1.07	108.15
	200	59.3 ± 1.03	

	50	37.83 ± 2.02	
Butanol Extract	100	52.79 ± 1.08	94.71
	200	68.86 ± 1.36	

	50	44.03 ± 1.41	
Ethanol extract	100	54.86 ± 1.28	91.07
	200	70.12 ± 1.17	

The data are expressed in mean ± S.E.M. *n* = 3 in each group.

**Table 3:** Total cholesterol (T-Ch), LDL cholesterol (LDL-c), HDL cholesterol (HDL-c); and triglycerides (TGs) in serum and liver of HFD-fed rats treated with EZA.

Groups	Control	HFD	HFD + fluv.	HFD + EZA
Serum (mmol/L)				

T-Ch	1.67 ± 0.20	2.72 ± 0.14*	1.77 ± 0.1^∗#^	1.74 ± 0.1^∗#^
TGs	0.96 ± 0.21	1.4 ± 0.16*	0.96 ± 0.07^∗#^	0.96 ± 0.04^∗#^
LDL-c	0.47 ± 0.22	1.61 ± 0.16*	0.49 ± 0.15^∗#^	0.53 ± 0.03^∗#^
HDL-c	1.02 ± 0.09	0.82 ± 0.04*	1.04 ± 0.07^#^	1.05 ± 0.06^#^

Liver (mg/100 mg WT)				

T-Ch	1.08 ± 0.05	2.45 ± 0.15*	1.22 ± 0.02^∗#^	1.27 ± 0.05^∗#^
TGs	0.63 ± 0.07	1.45 ± 0.11*	0.72 ± 0.06^∗#^	0.77 ± 0.04^∗#^
LDL-c	0.53 ± 0.03	1.78 ± 0.12*	0.45 ± 0.04^∗#^	0.48 ± 0.03 ^∗#^
HDL-c	0.44 ± 0.02	0.38 ± 0.01*	0.49 ± 0.02^∗#^	0.51 ± 0.02^∗#^

WT: wet tissue. Values are given as mean ± SD for groups of 8 animals each. Values are statistically presented as follows: *P < 0.05 significant differences compared to controls. ^#^ *P* < 0.05 significant differences compared to HFD rats.

**Table 4:** Serum electrolytes levels of control animals and HFD-fed rats. Values are given as mean ± SD for groups of 8 animals each.

Electrolytes	Control	HFD	HFD + fluv.	HFD + EZA
Na^+^ (mmol/L)	138.85 ± 0.75	129.8 ± 2.46*	134.85 ± 1.01^∗#^	137.92 ± 0.92^#@^
K^+^ (mmol/L)	4.38 ± 0.19	5.48 ± 0.12*	4.8 ± 0.13^∗#^	4.42 ± 0.18^#@^
Ca^2+^ (mmol/L)	2.86 ± 0.07	2.23 ± 0.07*	2.52 ± 0.07^∗#^	2.83 ± 0.03^#@^
Mg^2+^ (mmol/L)	1.16 ± 0.08	0.81 ± 0.05*	0.98 ± 0.03^∗#^	1.2 ± 0.06^#^
Cl^−^ (mmol/L)	111.15 ± 1.61	101.8 ± 1.67*	108.72 ± 0.85^∗#^	110.02 ± 1.76^#^

WT: wet tissue. Values are statistically presented as follows: *P < 0.05 significant differences compared to controls. ^#^ *P* < 0.05 significant differences compared to HFD rats ^@^ *P* < 0.05 significant differences to HFD rats treated with fluvastatin.

**Table 5:** Liver profile indices (AST, ALT, ALP, GGT, CPK, and T-Bili) and kidney parameters (creatinine, urea, and uric acid) of control and experimental groups of rats. Values are given as mean ± SD for groups of 8 animals each.

	Control	HFD	HFD + fluv.	HFD + EZA
Liver function				

AST (UI/L)	106.7 ± 11.52	194.42 ± 7.63*	128 ± 6.54^∗#^	123 ± 4.37^∗#^
ALT (UI/L)	60.57 ± 8.88	90.85 ± 5.75*	71 ± 9.14^∗#^	66.28 ± 5.4^#^
PAL (UI/L)	281.28 ± 42.8	627.42 ± 83.73*	365.28 ± 78.39^∗#^	308.28 ± 61.54^#^
GGT (UI/L)	3.42 ± 0.53	6.71 ± 0.75*	4.57 ± 0.74^∗#^	4.5 ± 1.04^∗#^
CPK (UI/L)	3128 ± 193	4147 ± 198*	3317 ± 185^#^	3439 ± 170^∗#^
T-Bili (*μ*mol/L)	1.66 ± 0.21	2.51 ± 0.24*	1.6 ± 0.33^#^	1.64 ± 0.26^#^

Kidney function				

Urea (mmol/L)	6.35 ± 0.41	8.47 ± 0.27*	6.41 ± 0.37^#^	6.79 ± 0.47^#^
Uric acid (*μ*mol/L)	62.73 ± 6.74	72.85 ± 6.6*	64.77 ± 7.46^#^	62.37 ± 5.14^#^
Creatinine (*μ*mol/L)	44.7 ± 0.94	64.38 ± 3.56*	41.3 ± 1.7^#^	46.4 ± 1.88^#^

Values are statistically presented as follows: ^∗^ *P* < 0.05 significant differences compared to controls. ^#^ *P* < 0.05 significant differences compared to HFD rats.
